# Genomic Epidemiology of SARS-CoV-2 Infection During the Initial Pandemic Wave and Association With Disease Severity

**DOI:** 10.1001/jamanetworkopen.2021.7746

**Published:** 2021-04-26

**Authors:** Frank P. Esper, Yu-Wei Cheng, Thamali M. Adhikari, Zheng Jin Tu, Derek Li, Erik A. Li, Daniel H. Farkas, Gary W. Procop, Jennifer S. Ko, Timothy A. Chan, Lara Jehi, Brian P. Rubin, Jing Li

**Affiliations:** 1 Center for Pediatric Infectious Disease, Cleveland Clinic Children’s, Cleveland, Ohio; 2 Robert J. Tomsich Pathology and Laboratory Medicine Institute, Cleveland Clinic, Cleveland, Ohio; 3 Department of Computer and Data Sciences, Case Western Reserve University, Cleveland, Ohio; 4 Center for Immunotherapy and Precision Immuno-Oncology, Lerner Research Center, Taussig Cancer Institute, Cleveland Clinic, Cleveland, Ohio; 5 Neurological Institute, Chief Research Information Office, Cleveland Clinic, Cleveland, Ohio

## Abstract

**Importance:**

Understanding of SARS-CoV-2 variants that alter disease outcomes are important for clinical risk stratification and may provide important clues to the complex virus-host relationship.

**Objective:**

To examine the association of identified SARS-CoV-2 variants, virus clades, and clade groups with disease severity and patient outcomes.

**Design, Setting, and Participants:**

In this cross-sectional study, viral genome analysis of clinical specimens obtained from patients at the Cleveland Clinic infected with SARS-CoV-2 during the initial wave of infection (March 11 to April 22, 2020) was performed. Identified variants were matched with clinical outcomes. Data analysis was performed from April to July 2020.

**Main Outcomes and Measures:**

Hospitalization, intensive care unit (ICU) admission, mortality, and laboratory outcomes were matched with SARS-CoV-2 variants.

**Results:**

Specimens sent for viral genome sequencing originated from 302 patients with SARS-CoV-2 infection (median [interquartile range] age, 52.6 [22.8 to 82.5] years), of whom 126 (41.7%) were male, 195 (64.6%) were White, 91 (30.1%) required hospitalization, 35 (11.6%) needed ICU admission, and 17 (5.6%) died. From these specimens, 2531 variants (484 of which were unique) were identified. Six different SARS-CoV-2 clades initially circulated followed by a rapid reduction in clade diversity. Several variants were associated with lower hospitalization rate, and those containing 23403A>G (D614G Spike) were associated with increased survival when the patient was hospitalized (64 of 74 patients [86.5%] vs 10 of 17 patients [58.8%]; χ^2^_1_ = 6.907; *P* = .009). Hospitalization and ICU admission were similar regardless of clade. Infection with Clade V variants demonstrated higher creatinine levels (median [interquartile range], 2.6 [−0.4 to 5.5] mg/dL vs 1.0 [0.2 to 2.2] mg/dL; mean creatinine difference, 2.9 mg/dL [95% CI, 0.8 to 5.0 mg/dL]; Kruskal-Wallis *P* = .005) and higher overall mortality rates (3 of 14 patients [21.4%] vs 17 of 302 patients [5.6%]; χ^2^_1_ = 5.640; *P* = .02) compared with other variants. Infection by strains lacking the 23403A>G variant showed higher mortality in multivariable analysis (odds ratio [OR], 22.4; 95% CI, 0.6 to 5.6; *P* = .01). Increased variants of open reading frame (ORF) 3a were associated with decreased hospitalization frequency (OR, 0.4; 95% CI, 0.2 to 0.96; *P* = .04), whereas increased variants of Spike (OR, 0.01; 95% CI, <0.01 to 0.3; *P* = .01) and ORF8 (OR, 0.03; 95% CI, <0.01 to 0.6; *P* = .03) were associated with increased survival.

**Conclusions and Relevance:**

Within weeks of SARS-CoV-2 circulation, a profound shift toward 23403A>G (D614G) specific genotypes occurred. Replaced clades were associated with worse clinical outcomes, including mortality. These findings help explain persistent hospitalization yet decreasing mortality as the pandemic progresses. SARS-CoV-2 clade assignment is an important factor that may aid in estimating patient outcomes.

## Key Points

**Question:**

Are SARS-CoV-2 variants, virus clades, or clade groups associated with disease severity and patient outcomes?

**Findings:**

In this cross-sectional study of 302 SARS-CoV-2 isolates, 6 different Global Initiative on Sharing All Influenza Data clades circulated in the community followed by a rapid reduction in clade diversity. Several variants, including 23403A>G (D614G), were significantly associated with lower hospitalization rates and increased patient survival.

**Meaning:**

These findings suggest that SARS-CoV-2 clade assignment is an important factor that may aid in estimating patient outcomes.

## Teaser

This cross-sectional study examines the association of identified SARS-CoV-2 variants, virus clades, and clade groups with disease severity and patient outcomes.

## Introduction

As of February 2021, there have been more 27 million confirmed SARS-CoV-2 infections in the US occurring in 3 waves.^1^ Before governmental policies aimed at infection containment were enacted, initial wave infections were travel related, most of which originated from Europe and were associated with high hospitalization and mortality rates in certain at-risk groups.^2,3^ Over time, disease associated with infection demonstrated decreasing length of stay and reduced case fatality ratios despite elevated numbers of hospitalizations.^4^ Although the development of antiviral medications and improved clinical care protocols have had substantial effects, the contribution of virus evolution on changes in clinical outcomes remains understudied.^5,6^

There are several nomenclature systems commonly used to classify SARS-CoV-2.^7,8,9,10^ Six distinct SARS-CoV-2 clades, in addition to the progenitor clade (Wuhan), are classified by the Global Initiative on Sharing All Influenza Data (GISAID): S, L, V, G, GH, and GR.^11^ These roughly correspond to the virus lineages A, B, B.2, B.1, B.1.*, and B.1.1.1, respectively.^8^ Three clades (G, GH, and GR) contain the 23403A>G (D614G) variant within the gene that encodes the spike glycoprotein. This variant is associated with increased infectivity and decreased clinical severity in several reports.^12,13^ Still, our understanding of disease severity associated with specific variants within different SARS-Cov-2 clades remains limited. In this cross-sectional study, we perform viral genome analysis through next-generation sequencing of SARS-CoV-2 clinical isolates that occurred during the initial 6 weeks of infection in Cleveland, Ohio. We matched identified variants and clades with disease severity and patient outcomes. Improved understanding of viral variants that alter disease outcomes are important for clinical risk stratification and may provide important clues to the complex virus-host relationship.

## Methods

A detailed description of the Cleveland Clinic COVID-19 Registry has been published previously^14^ (see eMethods in the Supplement). This study was approved by the Cleveland Clinic institutional review board and institutional biosafety committee. A waiver of consent was provided by the institutional review board for the use of residual samples. This study follows the Strengthening the Reporting of Observational Studies in Epidemiology (STROBE) reporting guideline for cross-sectional studies.^15^

### Specimen Selection

Specimens positive for SARS-CoV-2 by nucleic acid amplification performed at Cleveland Clinic Department of Laboratory Medicine from March 11 through April 22, 2020, were identified. Specimens with an indeterminate result,^16^ obtained from locations other than the nasopharynx, or with cycle threshold (C_T_) values greater than 30 were excluded. Poor quality sequencing reads occurred in specimens wherein the C_T_ was greater than 30 cycles (data not shown). Selection preference was given to specimens with C_T _of 26 cycles or fewer to ensure accuracy. Of 2334 positive specimens, 1750 (75.0%) had C_T _30 cycles or fewer. Of these, 302 (17.3%) isolates with representative sampling across the initial 6 weeks of SARS-CoV-2 circulation were selected.

### Library Preparation and Sequence Data Analysis

Total nucleic acid was purified from each specimen and subjected to reverse transcription, next-generation sequencing library preparation, sequencing, and data analysis according to the manufacturer’s recommendation (Paragon Genomics). Variants were called using the FreeBayes program version 1.1.0^17^ and were filtered at 5% and 10% allele fractions for insertion or deletion and single nucleotide variants, respectively (see eMethods in the Supplement). Genome coverage times 50 occurred in 97.6% of samples, with low coverage consistently observed at each end. Quality was ensured by monitoring mapping quality, phred score, and manual review of each variant for each sample.

### Phylogenetic Analysis

Genomic sequences were constructed for each isolate according to variants called from sequence reads and the reference sequence (NC_045512.2). Multiple sequence alignments were performed using MAFFT software version 7.0.^18^ A maximum likelihood approach in NextStrain^19^ was used to build the phylogenetic tree, and a local installation of Auspice from NextStrain was used to visualize the phylogenetic tree and associated meta data (see eMethods in the Supplement).

SARS-CoV-2 clade assignment followed GISAID clade guidelines and lineage nomenclature.^20^ Manual clade assignment was performed for isolates when clade defining variants frequency occurred below 90%. We further classified SARS-CoV-2 clades into 2 clade groups depending on the presence of the 23403A>G (D614G) spike glycoprotein variant. Clade group 1 included isolates without this variant (GISAID clades S, V, L, and Wuhan). Clade group 2 included isolates with this variant (GISAID clade groups G, GR, and GH).

### Statistical Analysis

#### Univariable Analysis

For clinical outcomes analysis, continuous variables were described using median and range; categorical variables were described using frequency and percentage. Demographic and clinical characteristics were compared between patients in different virus groups by using Kruskal-Wallis tests for continuous variables and Fisher exact or Pearson χ^2^ tests for categorical variables. All tests were 2-tailed, and significance was set at *P* < .05. PRISM statistical software version 8.4.3 (GraphPad Software) was used for all analyses.

#### Multivariable Analysis

To assess the association of demographic variables, comorbidity, clinical laboratory test results, and virus variant with clinical outcomes, we performed logistic regression analyses and built 3 different models for 2 different outcome variables: hospitalization and death, respectively. For each clinical variable, the 3 models are different in the way in which SARS-CoV-2 variants are incorporated into the model. For model 1, we included clade group as a binary variable. For model 2, we included the GISAID clade as a categorical variable. For model 3, we counted the total number of functional mutant alleles (including nonsynonymous single-nucleotide variants and insertions or deletions) within each of the 10 genes (S, E, M, N, OFR1ab, OFR3a, OFR6, OFR7a, OFR8, and OFR10) for each isolate, and treated each gene as 1 quantitative trait. Additionally, with hospitalization as the dependent variable, all the specimens were considered and we also included age, gender, race, smoking, and comorbidity for the following conditions: emphysema, asthma, diabetes, hypertension, coronary heart disease, heart failure, and immunosuppression. We separated data into training (80%) and testing (20%) for each model. We first built a full model using the training data by including all the variables by taking advantage of the StatsModels library in Python statistical software version 3.7 (Python).^21^ Because the sample size was limited, we first eliminated all the variables in the model whose coefficients have a *P* ≥ .30 (Wald test). We further iteratively eliminated variables on the basis of the *P* value of its coefficient (highest to lowest) until all the variables were below *P* ≤ .05. Specific variant variables (ie, clade group, clade assignment, and variants in genes) were added back to the final model if they were eliminated earlier. When we consider death as the dependent variable, we only included hospitalized samples, many of which had additional laboratory tests. We first performed missing data imputation on these variables using the IterativeImputer function in scikit-learn package in Python and converted each test into a binary variable: normal vs abnormal.^22^ Because the number of samples was much smaller and the number of variables was much greater compared with the clinical variable hospitalization, we first checked the number of samples in each category of a binary variable and eliminated those with fewer than 5 samples in any category. Linearly correlated variables were removed to leave 1 for each such group. We then removed variables in the full model with *P* > .5, followed by an iterative elimination of the least significant variable until all variables had coefficients with *P* < .05. The variant variables (ie, clade group, clade assignment, and variants in genes) were added back to the final model if they were eliminated earlier. Data analysis was performed from April to July 2020.

## Results

Virus-positive nasopharyngeal specimens from 302 patients (median [interquartile range [IQR] age, 52.6 [22.8-82.5] years) collected between March 11 and April 22, 2020, were selected for viral genome analysis. Median C_T_ value of selected specimens was 19.4 cycles (range, 13.2-30.0 cycles). Selected patients included 176 women (58.3%), 126 men (41.7%), 195 White individuals (64.6%), and 128 (42.4%) health care employees (Table 1). Ninety-one patients (30.1%) required hospitalization, of whom 35 (38.5% of admitted patients, 11.6% overall) required admission to the intensive care unit (ICU) and 17 died (18.7% of admitted patients, 5.6% overall).

**Table 1.** Patient Demographic Characteristics Between GISAID SARS-CoV-2 Clades and Clade Groups

Characteristic	All (N = 302)	GISAID clade, patients, No. (%)							Clade group, patients, No. (%)		
		Wu (n = 4)	S (n = 29)	V (n = 14)	G (n = 31)	GR (n = 23)	GH (n = 201)	*P* value	1 (n = 47)	2 (n = 255)	*P* value
Age, median (IQR), y	52.6 (22.8-82.5)	67.8 (59.8-75.8)	58.0 (43.4-81.6)	62.3 (52.4-72.1)	51.6 (23.0-80.2)	40.9 (15.3-66.5)	50.5 (19.9-81.0)	.05^a^	62.2 (39.5-73.0)	50.5 (20.6-80.0)	.002^a^
Health care employee											
Yes	128 (42.4)	2 (50.0)	7 (24.1)	2 (14.3)	14 (45.2)	12 (52.2)	91 (45.3)	.07	11 (23.4)	117 (45.9)	.004^a^
No	174 (57.6)	2 (50.0)	22 (75.9)	12 (85.7)	17 (54.8)	11 (47.8)	110 (54.7)		36 (76.6)	138 (54.1)	
Male	126 (41.7)	1 (25.0)	15 (51.7)	7 (50.0)	14 (45.2)	9 (40.9)	80 (39.6)	.77	23 (48.9)	103 (40.4)	.28
Race											
White	195 (64.6)	2 (50.0)	19 (65.5)	7 (50.0)	21 (67.7)	13 (54.5)	133 (66.3)	.75	28 (59.6)	167 (65.5)	.44
Black	67 (22.2)	2 (50.0)	4 (13.8)	7 (50.0)	5 (16.1)	3 (13.6)	46 (22.8)	.05	13 (27.7)	54 (21.2)	.33
Multiracial or Hispanic	8 (5.3)	0	4 (13.8)	0	1 (3.2)	3 (13.6)	8 (4.0)	.11	4 (8.5)	12 (4.7)	.29
Other^b^	14 (7.9)	0	2 (6.9)	0	4 (12.9)	4 (18.2)	14 (7.0)	.33	2 (4.3)	22 (8.6)	.32
Comorbidities											
Any smoking history	95 (31.5)	2 (50.0)	8 (27.6)	6 (42.9)	7 (22.6)	8 (34.8)	64 (31.8)	.71	16 (34.0)	79 (31.0)	.68
Any pulmonary condition^c^	62 (20.5)	2 (50.0)	7 (24.1)	4 (28.6)	7 (22.6)	8 (34.8)	34 (16.9)	.19	13 (27.7)	49 (19.2)	.19
Diabetes	54 (17.9)	2 (50.0)	4 (13.8)	1 (7.1)	9 (29.0)	4 (17.4)	34 (16.9)	.22	7 (14.9)	47 (18.4)	.57
Any cardiac condition^d^	115 (38.1)	3 (75.0)	8 (27.6)	7 (50.0)	12 (38.7)	6 (26.1)	79 (39.3)	.30	18 (38.3)	97 (38.0)	.96
Any immunosuppression^e^	67 (22.2)	2 (50.0)	5 (17.2)	6 (42.9)	9 (29.0)	2 (8.7)	43 (21.4)	.11	13 (27.7)	54 (21.2)	.33

Abbreviations: GISAID, Global Initiative on Sharing All Influenza Data; IQR, interquartile range.

^a^ Denotes* P* < .05 for comparison of median age of GISAID clades by ANOVA and between clade group by *t* test. Comparison of demographic data of GISAID clades and between clade group by χ^2^ analysis.

^b^ Includes Native American, Asian, or Pacific Islander.

^c^ Includes history of chronic obstructive pulmonary disease or asthma.

^d^ Includes history of hypertension, coronary artery disease, or heart failure.

^e^ Includes history of neoplastic disease, immunosuppressive disease, or use of immunosuppressive medications.

SARS-CoV-2 genomes of each patient specimen were sequenced and mapped against the reference Wuhan strain (Wuhan-Hu-1, NC_045512.2); 2531 variants (484 unique) were identified (eFigure 1 in the Supplement). The majority of variants (257 of 484 [53.1%]) were missense variants; silent variants were less common (157 of 484 variants [32.4%]). The study population demonstrated a median number of 5 variants per sample (range, 2-20 variants). Predominant variant locations included open reading frame 1 a/b (ORF1ab) (299 of 484 variants [61.8%]), spike glycoprotein (65 of 484 variants [13.4%]), nucleocapsid (32 of 484 variants [6.6%]), and ORF3a (20 of 484 variants [4.1%]). The most common nonsynonymous variants identified were 23403A>G (D614G spike) and 14408C>T (P323L ORF1ab).^23^ These 2 variants along with intergenic 241C>T (intergenic) and silent 3037C>T (F924 ORF1ab) variants had a coincident rate of 100% (eFigure 2 in the Supplement). Both common and rarely reported variants from the GISAID database were identified in our study population (eTable 1 in the Supplement).

**Figure 1.**
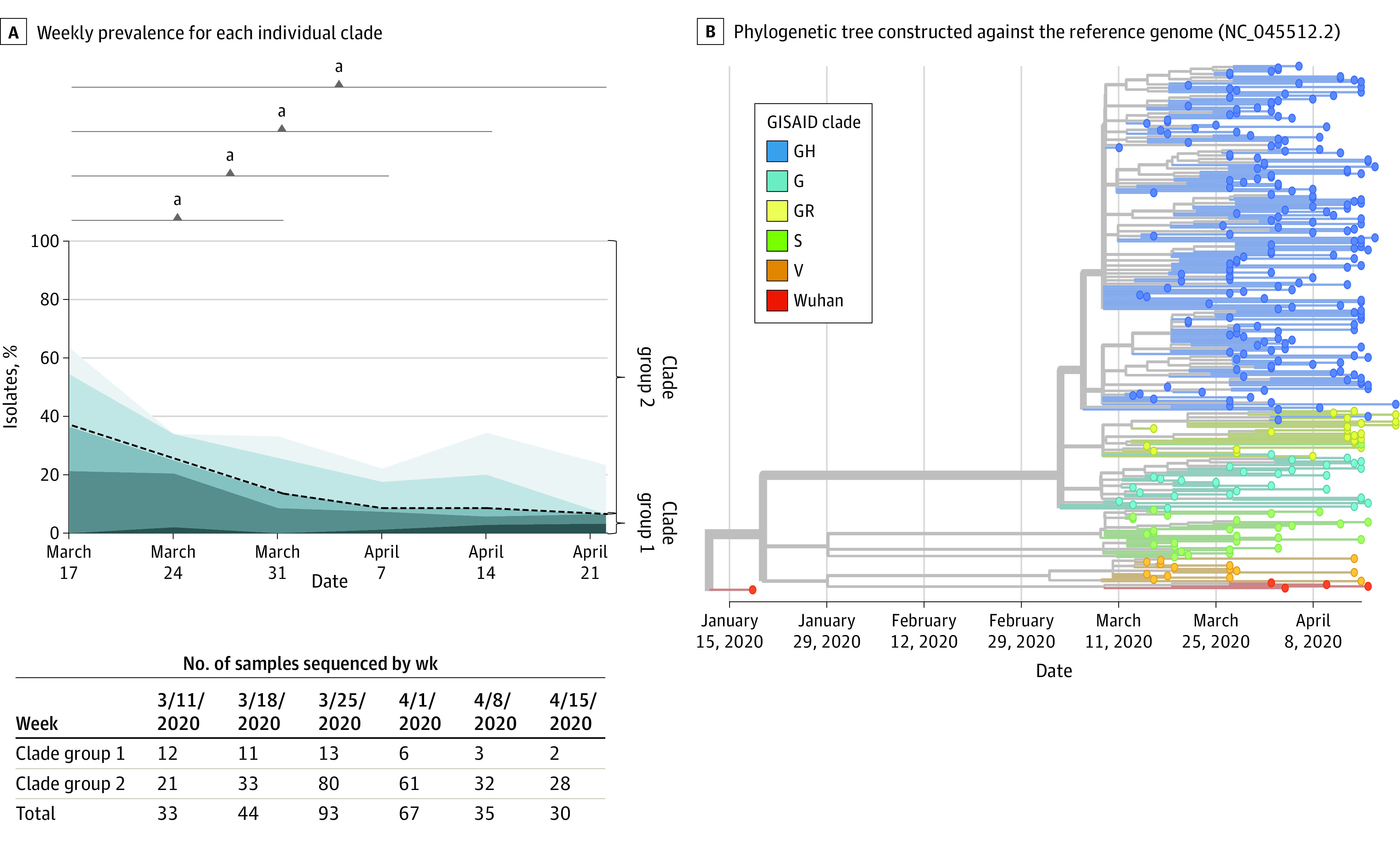
SARS-CoV-2 Clade Prevalence Over the Initial Pandemic Wave Genotypes of selected clinical samples were determined and categorized into Global Initiative on Sharing All Influenza Data (GISAID) clade. A, Weekly prevalence for each individual clade is displayed. GISAID clades were further clustered into 2 clade groups depending on the presence of the G614D spike glycoprotein variant (black dashed line). B, Phylogenetic tree constructed against the reference genome (NC_045512.2) using all samples. Timeline is displayed on the x-axis. The leaves are colored according to the GISAID clade, whereas the branches are labeled using NextStrain clade ID. The 2 systems are mostly consistent with each other. ^a^Comparison of clade group prevalence to the initial was performed by χ^2^ analysis at a significance level of *P* < .05.

After recognition of SARS-CoV-2 circulation in Cleveland on March 11, 2020, the 7-day rolling average of the initial pandemic wave peaked on April 11, 2020, then gradually declined. During this time, 6 different viral clades circulated; G, GR, and GH (clade group 2) represented 84.4% (255 of 302) of all identified isolates. The remainder (47 of 302 isolates [15.6%]) included V, S, and Wuhan clades (clade group 1). No isolates were identified belonging to clade group L. Patients in different clades showed differences in age (analysis of variance, *F* = 2.533; *P* = .046) with the Wuhan clade containing older patients (median [IQR] age, 67.8 [59.8-75.8] years) and GR the youngest (median [IQR] age, 40.8 [15.3-66.5] years). Patients infected with clade group 1 isolates were older (median [IQR] age, 62.2 [39.5-73.0] vs 50.5 [20.6-80.0] years; difference, 11.7 years; 95% CI, 9.7-13.7 years; *t* test, *P* = .002). No gender or racial differences were seen between the 2 main clade groups or within individual GISAID clades. During the initial weeks of the pandemic, there was a substantially higher prevalence of clade group 1 isolates. However, a rapid reduction in clade diversity was observed within 2 weeks of the start of SARS-CoV-2 testing (Figure 1). By the end of the study period, 90% of all circulating isolates (44 of 49 isolates) belonged to clade group 2. In total, there were 128 (42.3%) hospital employees included in this study. The difference in clade distribution between hospital employees and nonemployees was not significant (Table 1). However, nonemployees had a higher percentage of clade group 1 isolates compared with employees (36 of 174 nonemployees [20.7%] vs 11 of 128 employees [8.6%]; χ^2^_1_ = 8.186; *P* = .004).

Clinical outcomes were evaluated by variant and clade (eTable 2 in the Supplement and Table 2). No SARS-CoV-2 variants were associated with higher hospitalization rate. Several variants were associated with lower hospitalization rate, including 12809C>T (L4182F ORF1ab, 3 of 91 hospitalizations [3.3%] vs 22 of 211 hospitalizations [10.4%]; χ^2^_1_ = 4.215; *P* = .04) and 27964C>T (S24L ORF8, 0 of 91 hospitalizations [0%] vs 13 of 211 hospitalizations [6.2%]; χ^2^_1_ = 5.878; *P* = .01). Variants associated with clade group 2 (241C>T, 3037C>T, 14408C>T, and 23403A>G) were associated with increased patient survival when hospitalized (64 of 74 patients [86.5%] vs 10 of 17 patients [58.8%]; χ^2^_1_ = 6.907; *P* = .009). Frequency of hospitalization and ICU admission were similar regardless of clade. Clade V infection demonstrated higher mortality overall (3 of 14 deaths [21.4%] vs 17 of 302 deaths [5.6%]; χ^2^_1_ = 5.640; *P* = .02). Similarly, clade group 1 infection was associated with higher mortality than clade group 2 (7 of 47 deaths [14.9%] vs 10 of 255 deaths [3.9%]; χ^2^_1_ = 9.035; *P* = .002). Although no differences in viral load among GISAID clades were observed (eFigure 3 in the Supplement), clade V samples had lower viral loads (2.5 × 10^6^ vs 1.5 × 10^7^ copies/mL), whereas patients infected with clade group 2 had higher viral loads (1.6 × 10^7 ^vs 9.8 × 10^6^ copies/mL) than samples from other clades; however, the differences were not significant.

**Table 2.** SARS-CoV-2 Clade and Clade Group Prevalence in Hospitalization, ICU Admission, and Death

Variable	Patients, No. (%)					
	Nonhospitalized (n = 211)	Hospitalized				
		All (n = 91)	ICU (n = 35)	No ICU (n = 56)	Death (n = 17)	Survived (n = 74)
Clade						
Wu (n = 4)	1 (25.0)	3 (75.0)	1 (33.3)	2 (66.7)	1 (33.3)	2 (66.7)
S (n = 29)	19 (65.5)	10 (34.5)	3 (30.0)	7 (70.0)	3 (30.0)	7 (70.0)
V (n = 14)	10 (71.4)	4 (28.6)	3 (75.0)	1 (25.0)	3 (75.0)^a^	1 (25.0)
G (n = 31)	18 (58.1)	13 (41.9)	4 (30.8)	9 (69.2)	0	13 (100.0)
GR (n = 23)	18 (78.3)	5 (21.7)	2 (40.0)	3 (60.0)	0	5 (100.0)
GH (n = 201)	146 (72.1)	56 (27.9)	22 (39.3)	36 (64.3)	10 (17.9)	46 (82.1)
Clade group						
1 (n = 47)	30 (63.8)	17 (36.2)	7 (41.2)	10 (58.8)	7 (41.2)^b^	10 (58.8)
2 (n = 255)	181 (71.0)	74 (29.0)	28 (37.8)	48 (64.9)	10 (13.5)	64 (86.5)
Total (N = 302)	211 (69.9)	91 (30.1)	35 (38.5)	58 (63.7)	17 (18.7)	74 (81.3)

Abbreviation: ICU, intensive care unit.

^a^ *P* = .004; χ^2^_1_ = 8.143.

^b^ *P* = .009; χ^2^_1_ = 6.907.

**Figure 2.**
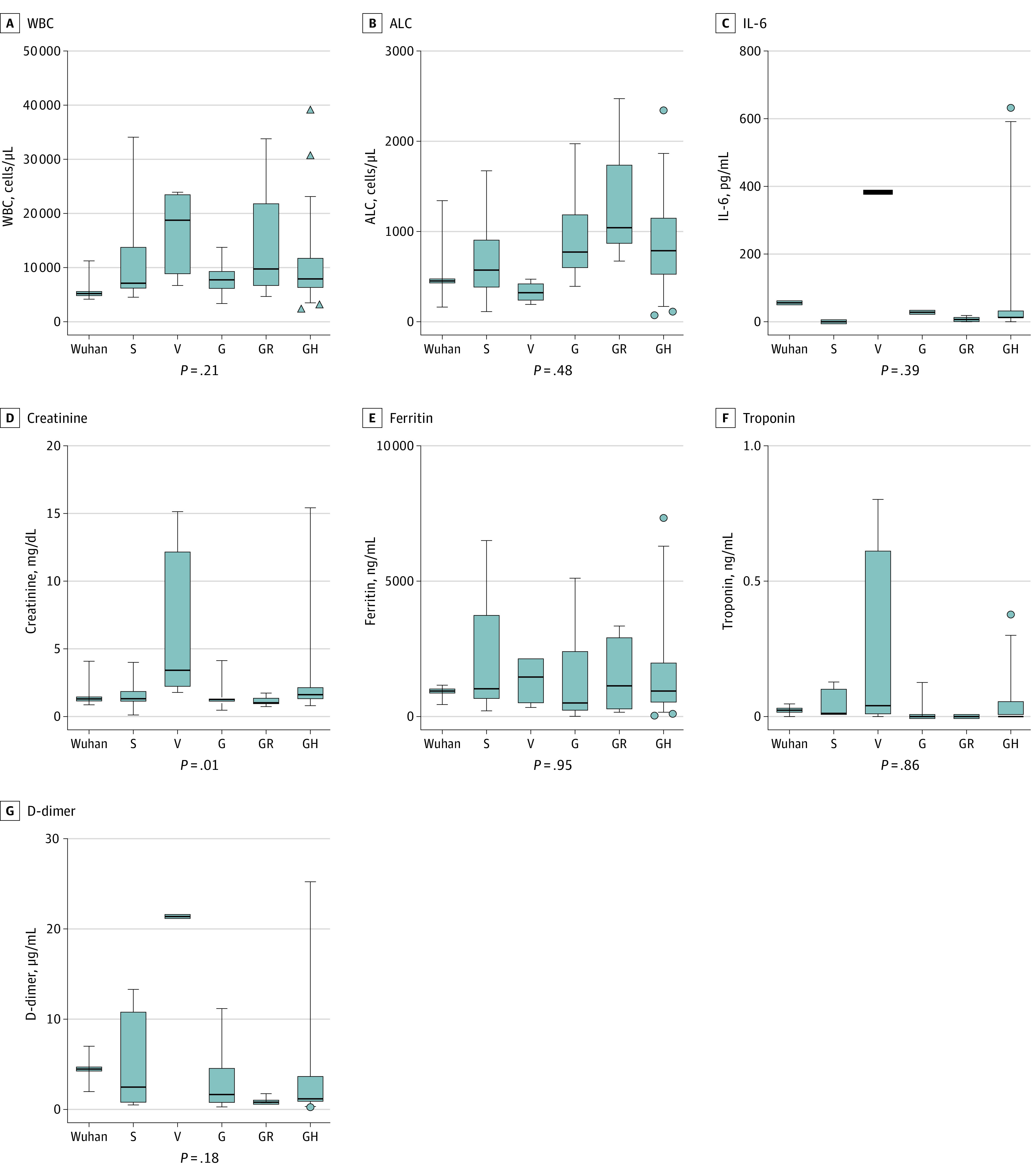
Comparison of Laboratory Abnormalities Among Different SARS-CoV-2 Clades Box and whiskers plot display first through 99th percentile laboratory results among patients infected with specific SARS-CoV-2 clades. *P* values for ordinary 1-way analysis of variance was performed at a significance level of *P* < .05. ALC indicates absolute lymphocyte count; IL-6, interleukin-6; WBC, white blood cell count. SI conversion factors: To convert ALC to cells times 10^9^ per liter, multiply by 0.001; creatinine to micromoles per liter, multiply by 88.4; D-dimer to nanomoles per liter, multiply by 5.476; ferritin to micrograms per liter, multiply by 1.0; white blood cell count to cells times 10^9 ^per liter, multiply by 0.001.

Patient laboratory values were compared among SARS-CoV-2 clades (Figure 2). Significant variation was observed for interleukin-6, creatinine, and D-dimer among individual variants (eFigure 4 in the Supplement). With the exception of creatinine, no variation in white blood cell count, absolute lymphocyte count, interleukin-6, ferritin, troponin, or D-dimer among GISAID clades was seen. Patients with clade V infection had significantly higher creatinine values than patients infected with other clades (median [IQR], 2.6 [−0.4 to 5.5] mg/dL vs 1.0 [0.2 to 2.2] mg/dL; mean creatinine difference, 2.9 mg/dL [95% CI, 0.8 to 5.0 mg/dL]; Kruskal-Wallis *P* = .005) (to convert creatinine to micromoles per liter, multiply by 88.4). No significant variation of laboratory studies was observed between clade groups (eFigure 5 in the Supplement).

When all variables were evaluated together, including variants using multivariable logistic regression, both age and male sex increased the risk of hospitalization for all 3 models (Table 3). Neither clade group (model 1) nor individual clade (model 2) was significantly associated with hospitalization. Additionally, history of coronary heart disease was not significant in these models. For variants in SARS-CoV-2 genes (model 3), increasing variant within ORF3a was associated with a decreased risk of hospitalization (odds ratio [OR], 0.4; 95% CI, 0.2 to 0.96; *P* = .04). Infection by strains lacking the 23403A>G variant showed higher mortality in multivariable analysis (OR, 22.4; 95% CI, 0.6 to 5.6; *P* = .01). For mortality, both model 1 and model 2 identified age, immunosuppression, and abnormal creatinine level (>1.22 mg/dL) to be significantly associated with increased mortality. Clade group 1 was significantly associated with an increased risk of death (model 1). Although individual clades (model 2) have consistent direction (positive or negative) with the clade group (model 1), they were not statistically significant because of limited sample size in some clades. Increased Spike (OR, 0.01; 95% CI, <0.01 to 0.3; *P* = .01) and ORF8 (OR, 0.03; 95% CI, <0.01 to 0.6; *P* = .03) variants significantly increased survival (model 3).

**Table 3.** Logistic Regression Results Using Hospitalization or Death as Dependent Variables

Variables	Model 1		Variables	Model 2		Variables	Model 3	
	OR (95% CI)	*P* value		OR (95% CI)	*P* value		OR (95% CI)	*P* value
Hospitalization as the dependent variable								
Age	1.1 (1.05-1.1)	<.01	Age	1.1 (1.1-1.1)	<.01	Age	1.1 (1.1-1.1)	<.01
Male	2.4 (1.1-5.0)	.02	Male	2.5 (1.2-5.2)	.02	Male	2.7 (1.3-5.7)	.01
Coronary heart disease	3.0 (0.8-8.6)	.06	Coronary heart disease	2.9 (0.94-8.9)	.06	ORF1ab	0.9 (0.7-1.2)	.40
Clade group (1)	0.8 (0.3-2.0)	.60	Clade S	0.3 (0.01-17.7)	.60	Spike	1.0 (0.5-2.1)	>.99
			Clade V	0.2 (<0.01-14.9)	.50	ORF3a	0.4 (0.2-0.96)	.04
			Clade G	1.0 (0.02-59.8)	>.99	ORF7a	0.5 (0.04-7.0)	.60
			Clade GR	0.2 (<0.01-16.4)	.50	ORF8	1.2 (0.5-2.9)	.70
			Clade GH	0.3 (0.01-15.0)	.50	Nucleocapsid	0.6 (0.3-1.2)	.10
LLR *P* value		9.5 × 10^−15^			8.7 × 10^−14^			5.9 × 10^−13^
Accuracy, %	80.2			78.0			82.4	
Death as the dependent variable								
Age	1.2 (1.1-1.4)	<.01	Age	1.2 (1.1-1.4)	<.01	Age	1.3 (1.1-1.6)	<.01
Any immunosuppression	18.9 (1.7-212.9)	.02	Any immunosuppression	25.5 (1.7-373.9)	.02	ORF1ab	1.7 (0.7-4.2)	.20
Creatinine level >1.22 mg/dL	18.7 (1.5-226.4)	.02	Creatinine level >1.22 mg/dL	18.7 (1.4-253.7)	.03	Spike	0.01 (<0.01-0.3)	.01
Clade group (1)	22.4 (1.9-269.9)	.01	Clade S	3.9 (0.1-283.2)	.50	ORF3a	0.2 (0.01-5.4)	.30
			Clade V	4.9 (0.03-961.0)	.60	ORF8	0.03 (<0.01-0.6)	.03
			Clade G	0.3 (<0.01-768.2)	.80	Nucleocapsid	0.6 (0.04-9.1)	.70
			Clade GH	0.1 (<0.01-6.7)	.30			
LLR *P* value		2.7 × 10^−8^			7.1 × 10^−7^			5.1 × 10^−7^
Accuracy, %	89.3			89.3			78.6	

Abbreviations: LLR, log likelihood ratio; OR, odds ratio.

SI conversion factor: To convert creatinine to micromoles per liter, multiply by 88.4.

## Discussion

There is an ever-increasing amount of SARS-CoV-2 genomic data being deposited in national and international sequencing databases.^20^ Similar to our findings, prevalent variants include 23403A>G (D614G Spike), 14408C>T (P323L ORF1ab), and 25563G>T (Q57H ORF3a).^24^ Still, our understanding of clinical differences associated with viral clade or specific variants remains limited. Reports show that strains containing D614G had higher viral loads in patient specimens, yet no difference in hospitalization outcomes.^12,13,25^ Other variants associated with altered severity are sparsely reported.^26^ Still, most investigations have found no significant difference in outcomes of hospitalization or death among major clades.^7,27^ One explanation for these findings is that many clinical studies on SARS-CoV-2 occur when the genetic diversity within a community has diminished. Often, D614G genotype strains are disproportionately represented, impacting the ability to discern differences between clades in smaller studies.^28,29^ Here, we describe a large investigation correlating clinical outcomes as a function of first-wave genotypes.

The Cleveland Clinic was among the first hospital systems in the US to provide community screening for SARS-CoV-2, offering a unique perspective of early virus dynamics. With the exception of a slight female predominance, our analysis is a representative sampling of the thousands of patients during the first wave of infection in Cleveland, Ohio.^16^ SARS-CoV-2–infected patients tended to be older, have cardiac and pulmonary comorbidities, and have a higher representation among socioeconomically disadvantaged racial/ethnic groups compared with the community. We found that the highest genomic diversity of SARS-CoV-2 occurred during the initial weeks, when 5 of the 6 described GISAID clades in addition to isolates closely resembling the reference Wuhan strain circulated. Such early diversity is consistent with the interpretation that multiple SARS-CoV-2 infection events occurred in this community through repeated introduction of viruses from Asia, Europe, and elsewhere within the US.

Clade group 2 contains the D614G variant and has been associated with increased infectivity in several reports.^30^ It has been hypothesized that the resultant amino acid change alters electrostatic interactions of viral protein subunits, leading to a more fusogenic ligand and enabling more efficient binding to the angiotensin converting enzyme 2 receptor.^7,31,32^ Many epidemiological investigations have demonstrated that this variant rapidly becomes the dominant form in a community following its introduction.^33^ However, although these reports are based on analysis of sequence submissions to international databases, our data provide a robust analysis of SARS-CoV-2 clade dynamics within a fixed community. The prevalence of clade group 2 rapidly increased in our community within weeks despite both clades being established. This suggests that clade group 2 has a fitness advantage over clade group 1. State and federal responses may have augmented the prevalence of clade group 2 through prevention of continued introduction of new clades from outside the community and thereby decreased overall mortality.

No specific viral variants were associated with increased hospitalization frequency in our cohort; however, several variants were associated with lower hospitalization rates, all occurring in viruses of clade GH. Similarly, we found no significant difference among SARS-CoV-2 clades for hospitalization and ICU admission, but differences in mortality were identified. Clade group 1 and specifically clade V were significantly associated with increased mortality in univariable and multivariable analysis. The multivariable models also demonstrated that accrued variants in spike and ORF8 were associated with decreased mortality, whereas accumulated changes in ORF3a were associated with decreased hospitalization. Surprisingly, the ORF1ab gene was not linked to either hospitalization or mortality in multivariable analysis despite containing the largest number of identified variants. Viral load was also not significantly different between clade groups, and loads in clade V specimens were lower, contrary to reports that higher viral load is associated with increased disease severity.^34,35^ Our findings demonstrate that the continued evolution of SARS-CoV-2 leads to less virulence. Given that our study period was during the initial weeks of the pandemic, it is unlikely that differences in survival were due to differences in patient care protocols, limitations of supplies or equipment, ICU bed space availability, or the use of antiviral medications.

Clade V is hallmarked by 2 nonsynonymous variants, 11083G>T (L37F ORF1ab) and 26144G>T (G251V ORF3a), leading to alterations in the NSP6 and NS3 proteins, respectively. Although the clinical implications of these variants remain unclear, 1 study^36^ noted that the 11083G>T variant was associated with asymptomatic transmission. However, the 26144G>T variant has been associated with epitope loss due to decreased protein flexibility, which may influence pathogenesis through antibody escape.^37^ In addition, this variant is thought to have dramatically attenuated binding affinity.^38^ Finally, infection with clade group V was associated with significantly higher creatinine values compared with other SARS-CoV-2 clades. Kidney injury has been associated with increased mortality in previous studies.^39,40^ This finding suggests that clade may have a specific predisposition for kidney involvement. Additional studies comparing SARS-CoV-2 genotypes in patients with and without kidney dysfunction are warranted.

### Limitations

Our study had several limitations owing to the smaller number of isolates from clade group 1, including clade V, which contains 14 patients. Additionally, our sampling paralleled the community outbreak where most patients did not require hospitalization or ICU care and mortality was infrequent. Together, this adversely affects the power to discern outcomes from underrepresented clades. Further analysis focusing on patients from the initial pandemic wave and targeting isolates from clade group 1 (Wuhan, S, and V), in addition to expanding virus genotyping of patients with higher severity of disease, should be performed to further clarify the clinical differences among clades. In addition, we combined neoplastic disease within the immunosuppression group. There is now growing understanding that SARS-CoV-2 outcomes in patients with neoplastic disease is far different than those receiving immunosuppression therapy. Further analysis examining the effect of virus clade on severity within these groups should be performed separately.

## Conclusions

This cross-sectional study demonstrates a dynamic shift in SARS-CoV-2 clade diversity occurring very early in the pandemic following introduction into Cleveland, Ohio. Within weeks of SARS-CoV-2 testing, we found a profound shift toward clade group 2 genotypes. The replaced clades (Wuhan, S, and V) were associated with higher mortality. Accrued variants in spike, ORF8, and ORF3a were associated with improved clinical outcomes. These findings are consistent with the observation of persistent hospitalization yet decreasing mortality as the pandemic progresses. SARS-CoV-2 clade assignment is an important factor in algorithms that may be used to estimate patient outcomes.

## Notes

Supplement. **eMethods.** **eReferences.** **eFigure 1.** Overview and Distribution of Identified SARS-CoV-2 Variants **eFigure 2. **Distribution of Single-Nucleotide Variants Across SARS-CoV-2 Genome **eTable 1.** Common Variants Identified During Initial Wave of SARS-CoV-2 Pandemic **eTable 2.** Prevalence of Select SARS-CoV-2 Variants in Hospitalization, ICU Admission, and Death **eFigure 3.** SARS-CoV-2 Viral Load by GSAID Clade and Clade Group **eFigure 4.** Specific Laboratory Abnormalities Among Selected SARS-CoV-2 Variants **eFigure 5. **Patient Laboratory Anomalies Between Different SARS-CoV-2 Clade Groups
