# Supplementary material for: Genomic Epidemiology of SARS-CoV-2 Infection During the Initial Pandemic Wave and Association With Disease Severity
Source: JAMA Netw Open. 2021 Apr 26;4(4):e217746. doi: 10.1001/jamanetworkopen.2021.7746 (PMC8076962; doi:10.1001/jamanetworkopen.2021.7746)
Supplement: Supplement. — eMethods. eReferences. eFigure 1. Overview and Distribution of Identified SARS-CoV-2 Variants eFigure 2. Distribution of Single-Nucleotide Variants Across SARS-CoV-2 Genome eTable 1. Common Variants Identified During Initial Wave of SARS-CoV-2 Pandemic eTable 2. Prevalence of Select SARS-CoV-2 Variants in Hospitalization, ICU Admission, and Death eFigure 3. SARS-CoV-2 Viral Load by GSAID Clade and Clade Group eFigure 4. Specific Laboratory Abnormalities Among Selected SARS-CoV-2 Variants eFigure 5. Patient Laboratory Anomalies Between Different SARS-CoV-2 Clade Groups [file jamanetwopen-e217746-s001.pdf]

Supplemental Online Content

Esper FP, Cheng YW, Adhikari TM, et al. Genomic epidemiology of SARS-CoV-2 infection during the initial pandemic wave and association with disease severity. *JAMA Netw Open*. 2021;4(4):e217746. doi:10.1001/jamanetworkopen.2021.7746

eMethods.

eReferences.

eFigure 1. Overview and Distribution of Identified SARS-CoV-2 Variants

eFigure 2. Distribution of Single-Nucleotide Variants Across SARS-CoV-2 Genome

eTable 1. Common Variants Identified During Initial Wave of SARS-CoV-2 Pandemic

eTable 2. Prevalence of Select SARS-CoV-2 Variants in Hospitalization, ICU Admission, and Death

eFigure 3. SARS-CoV-2 Viral Load by GSAID Clade and Clade Group

eFigure 4. Specific Laboratory Abnormalities Among Selected SARS-CoV-2 Variants

eFigure 5. Patient Laboratory Anomalies Between Different SARS-CoV-2 Clade Groups

This supplemental material has been provided by the authors to give readers additional information about their work.

© 2021 Esper FP et al. *JAMA Network Open.*

eMethods:

Library preparation and sequence data analysis

Briefly, complementary deoxyribonucleic acid (cDNA) synthesis via RT was performed in 20 µL reactions (10 minutes at 8°C and 80 minutes at 42°C). The derived panel of 343 amplicons utilized for SARS-CoV-2 enrichment covered 99.7% of the viral genome (MN908947/NC_045512.2) with 92 bases uncovered at each end of the genome. Purified cDNA was subject to multiplex PCR (10 minutes at 95°C, followed by 10 cycles at 98 °C for 15 seconds each and 60 °C for 5 minutes). Excess primers and oligos were subsequently removed from the purified PCR products, after which a second round of PCR to append indexing primers was performed (initial denaturation, 10 minutes at 95°C, followed by 24 cycles of 98°C for 15 seconds and 60°C for 75 seconds).

Sequencing libraries were prepared and quality was assessed visually using an Agilent® 2100 Bioanalyzer® (Agilent, Santa Clara CA). The presence of a ~275 bp peak indicated successful amplification and these libraries were sequenced using a MiSeq instrument (Illumina, San Diego, CA).

Raw fastq reads were extracted by Illumina bcl2fastq (v2.20.0) and mapped to the reference genome Wuhan-Hu-1 (NC_045512.2) using BWA program (1). GISAID genome data were used to compile minor allele frequency results on June, 1, 2020 with total 35,278 sequences selected. Only those sequences classified as high coverage or complete were utilized. Variants were called using FreeBayes program (2) and filtered at 5% and 10% allele fractions for insertion or deletion (INDEL) and single nucleotide variants (SNV), respectively. Amino acid changes were annotated using snpEff (v4.5) program (3). All variant data were visually examined in Integrative Genome

© 2021 Esper FP et al. *JAMA Network Open.*

Browser to eliminate artifacts. Sequences were deposited into GISAID with Accession IDs: EPI_ISL_943808, EPI_ISL_955982 to EPI_ISL_956269, and EPI_ISL_983331 to EPI_ISL_983343.

SARS-CoV-2 Copy number determination

Virus quantification was carried out by mixing total human RNA with various copies of the synthetic Covid-19 RNA to generate a standard calibration curve. The viral load (copy number) in each clinical specimen was then derived from the Ct value using the standard curve.

Clinical Outcomes

Clinical outcomes were obtained from the Cleveland Clinic COVID19 registry (4). This registry includes demographic, laboratory, and clinical data from patients tested for SARS-CoV-2 at Cleveland Clinic. Registry variables were chosen to reflect available literature on COVID-19 disease characterization, progression, and proposed treatments and included demographics, co-morbidities, medications, presenting symptoms, treatment, and disease outcomes. Capture of detailed research data was facilitated by the creation of standardized clinical templates implemented across the healthcare system as patients were seeking care for COVID-19-related concerns. Data were extracted via previously validated automated feeds from electronic health records (5) (EPIC; EPIC Systems Corporation) and manually by a study team trained on uniform sources for the study variables. Study data were collected and managed using REDCap electronic data capture tools hosted at the Cleveland Clinic (6, 7). Aberrant laboratory values, occurring within 21 days of hospitalization, were manually collected.

NextStrain Phylogenetic analysis

In this analysis, we used the NextStrain version nextstrain.cli 2.0.0.post1. For this analysis, we focused on performing multiple sequence alignment and constructing the phylogenetic tree.

© 2021 Esper FP et al. *JAMA Network Open.*

Nextstrain uses MAFFT version 7.0 and IQTree version 2.1.2 in the backend to perform multiple sequence alignment and to build the phylogenetic tree respectively (8, 9). During the multiple sequence alignment, we used all the default parameters that include: 6merpair algorithm for distance calculation, *weighti* 2.7 algorithm to calculate the consistency term from pairwise alignment, fast fourier transform (FFT) algorithm to identify the homologous regions in group-to-group alignment, PartTree algorithm for tree partition with 50 as the number of partitions, 1.53 as the penalty for opening a gap and 0.123 as the penalty for extending a gap in group-to-group alignment and BLOSUM62 as the substitution matrix to assign a score for aligning pairs.

In the process of building the phylogenetic tree, we used iqtree which creates phylogenetic trees using maximum likelihood method with default parameters. These default parameters include, generalized time reversible model (GTR) to build the tree with 4 cpu cores, 50 initial parsimony trees to implement randomized stepwise addition, subtree pruning and regafting, 4 total iterations to optimize the result and 0.05 of log likelihood epsilon for final model parameter estimation along with other default configurations present in NextStrain (10). In this experiment, all samples (n=302) were ultimately selected for phylogenetic tree building using NC_045512.2 genome as our reference sequence.

eReferences

1. Li H, Durbin R. Fast and accurate short read alignment with Burrows-Wheeler transform. Bioinformatics. 2009;25(14):1754-60.
2. Erik Garrison GM. Haplotype-based variant detection from short-read sequencing. arXiv. 2012;arXiv:1207.3907v2.
3. Cingolani P, Platts A, Wang le L, Coon M, Nguyen T, Wang L, et al. A program for annotating and predicting the effects of single nucleotide polymorphisms, SnpEff: SNPs in the genome of Drosophila melanogaster strain w1118; iso-2; iso-3. Fly (Austin). 2012;6(2):80-92.
4. Jehi L, Ji X, Milinovich A, Erzurum S, Rubin BP, Gordon S, et al. Individualizing Risk Prediction for Positive Coronavirus Disease 2019 Testing: Results From 11,672 Patients. Chest. 2020;158(4):1364-75.
5. Milinovich A, Kattan MW. Extracting and utilizing electronic health data from Epic for research. Ann Transl Med. 2018;6(3):42.

© 2021 Esper FP et al. *JAMA Network Open.*

6. Harris PA, Taylor R, Thielke R, Payne J, Gonzalez N, Conde JG. Research electronic data capture (REDCap)--a metadata-driven methodology and workflow process for providing translational research informatics support. J Biomed Inform. 2009;42(2):377-81.
7. Harris PA, Taylor R, Minor BL, Elliott V, Fernandez M, O'Neal L, et al. The REDCap consortium: Building an international community of software platform partners. J Biomed Inform. 2019;95:103208.
8. Katoh K, Standley DM. MAFFT multiple sequence alignment software version 7: improvements in performance and usability. Mol Biol Evol. 2013;30(4):772-80.
9. Nguyen LT, Schmidt HA, von Haeseler A, Minh BQ. IQ-TREE: a fast and effective stochastic algorithm for estimating maximum-likelihood phylogenies. Mol Biol Evol. 2015;32(1):268-74.
10. Hadfield J, Megill C, Bell SM, Huddleston J, Potter B, Callender C, et al. Nextstrain: real-time tracking of pathogen evolution. Bioinformatics. 2018;34(23):4121-3.

© 2021 Esper FP et al. *JAMA Network Open.*

eFigure 1 . Overview and Distribution of Identified SARS-CoV-2 Variants

**Total unique variants identified**	484
**Variant Occurrence in Study Population**	
>100	6 (1.2%)
11 - 100	23 (4.8%)
2-10	100 (20.7%)
1	355 (73.3%)
**Variant Type**	
Missense_variant nonsynonmous	257 (53.1%)
Silent_variant synonymous	157 (32.4%)
Intergenic variant	19 (3.9%)
Frameshift deletion	17 (3.5%)
Inframe deletion	15 (3.1%)
Nonsense_variant nonsynonmous	11 (2.3%)
Inframe delins	1 (0.2%)
Frameshift insertion	1 (0.2%)
Frameshift delins	1 (0.2%)
Nonsense_variant stop loss	1 (0.2%)

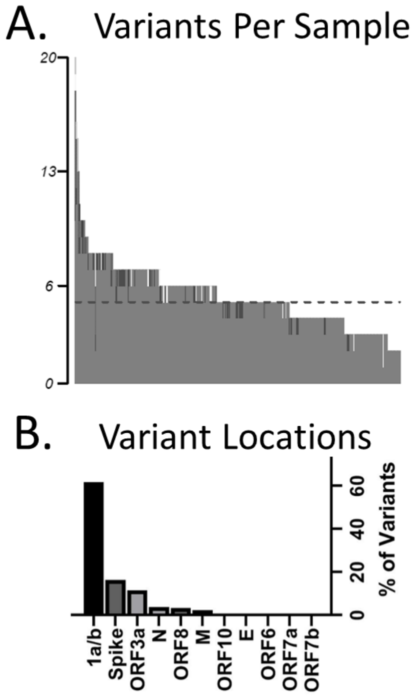

A) Number of variants identified by sample. B) Percent of identified variants by SARS-CoV-2 gene location.

© 2021 Esper FP et al. *JAMA Network Open.*

eFigure 2. Distribution of Single nucleotide variants across SARS-CoV-2 genome

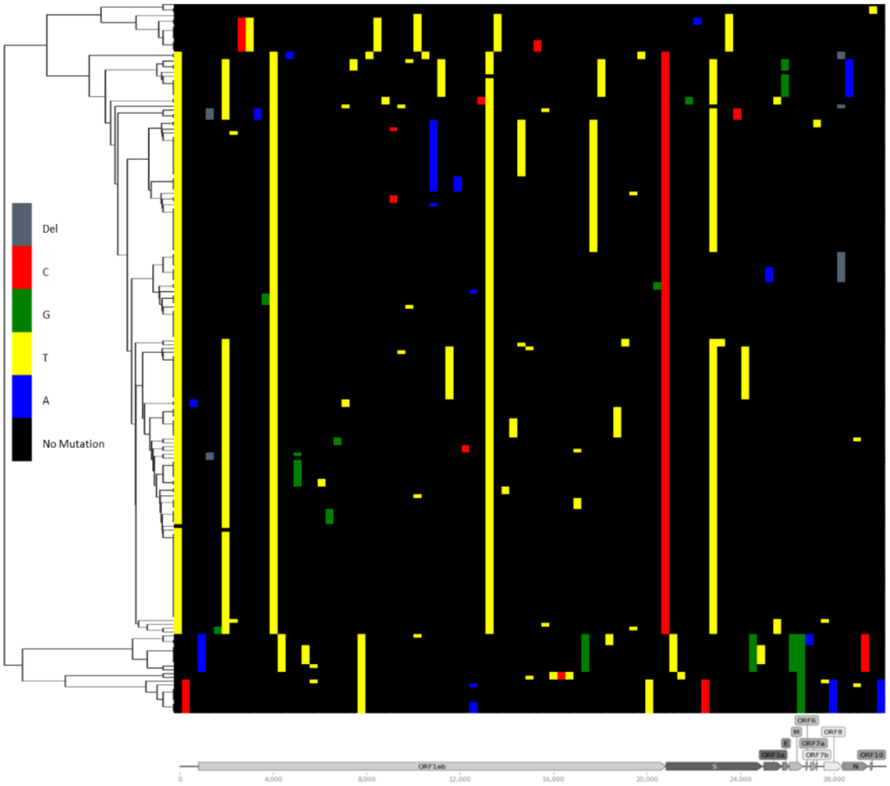

eFigure 2. Distribution of Single-Nucleotide Variants Across SARS-CoV-2 Genome. Each row is one sample and each column is one genomic location with at least two mutations. The samples are clustered based on the UPGMA algorithm.

© 2021 Esper FP et al. *JAMA Network Open.*

eTable 1: Common Variants Identified During Initial Wave of SARS-CoV-2 Pandemic				
**Variant**	**Gene**	**Effect**	**MAF**	**# of Samples**
241C>T	Intergenic	Intergenic variant	0.77	261
3037C>T	ORF1ab	Silent	0.74	261
14408C>T	ORF1ab	P4715L	0.75	261
23403A>G	Spike	N614G	0.74	261
25563G>T	ORF3a	G57H	0.16	207
1059C>T	ORF1ab	T265I	0.15	147
18877C>T	ORF1ab	Silent	0.01	58
28144T>C	ORF8	L84S	0.13	31
8782C>T	ORF1ab	Silent	0.13	30
12809C>T	ORF1ab	L4182F	0.0008	28
26625C>T	Matrix	Silent	0.002	27
11878C>A	ORF1ab	Silent	0.02	26
28881G>A	Nucleocapsid	R203K	0.03	24
15237C>T	ORF1ab	Silent	0.0008	20
361A>G	ORF1ab	Silent	0.03	19
22468G>T	Spike	Silent	0.0008	19
28878G>A	Nucleocapsid	S202N	0.0007	19
29742G>A	Intergenic	Intergenic variant	0.0007	19
25559T>G	ORF3a	F56C	0.001	18
11083G>T	ORF1ab	p.Leu3606Phe	0.067	17
11916C>T	ORF1ab	S3884L	0.009	16
14805C>T	ORF1ab	Silent	0.07	14
18998C>T	ORF1ab	A6245V	0.005	14
26144G>T	ORF3a	G251V	0.05	14
29540G>A	Intergenic	Intergenic variant	0.01	14
27964C>T	ORF8	S24L	0.01	13

© 2021 Esper FP et al. *JAMA Network Open.*

eTable 2. Prevalence of Select SARS-CoV-2 Variants in Hospitalization, ICU Admission, and Death

Gene and variant	Variants, No. (%)					
	**Hospitalized (N=91)**	**Non-Hospitalized (N=211)**	**Hospitalized: ICU (N=35)**	**Hospitalized: No ICU (N=56)**	**Hospitalized: Death (N=17)**	**Hospitalized: Survived (N=74)**
ORF1ab						
241C>T	74 (81.3)	181 (85.8)	28 (80.0)	48 (85.7)	10 (58.8)	64 (86.5)*
1059C>T	35 (38.5)	107 (50.7)	13 (37.1)	24 (42.9)	6 (35.3)	29 (39.2)
2480A>G	4 (4.4)	8 (3.8)	3 (8.6)	1 (1.8)	3 (17.6)	1 (1.4)*
2558C>T	4 (4.4)	8 (3.8)	3 (8.6)	1 (1.8)	3 (17.6)	1 (1.4)*
3037C>T	74 (81.3)	181 (85.8)	28 (80.0)	48 (85.7)	10 (58.8)	63 (86.5)*
8782C>T	9 (9.9)	19 (9.0)	3 (8.6)	6 (10.7)	3 (17.6)	6 (8.1)
9170C>T	4 (4.4)	8 (3.8)	3 (8.6)	1 (1.8)	3 (17.6)	1 (1.4)*
11083G>T	3 (3.3)	13 (6.2)	2 (5.7)	1 (1.8)	3 (17.6)	0 (0.0)*
11878C>A	10 (11.0)	16 (7.6)	4 (11.4)	6 (10.7)	1 (5.9)	9 (12.2)
11916C>T	2 (2.2)	13 (6.2)	1 (2.9)	1 (1.8)	1 (5.9)	1 (1.4)
12809C>T	3 (3.3)	22 (10.4)*	2 (5.7)	2 (3.6)	1 (5.9)	2 (2.7)
14408C>T	74 (81.3)	181 (85.8)	28 (80.0)	48 (85.7)	10 (58.8)	64 (86.5)*
14805C>T	4 (4.4)	10 (4.7)	3 (8.6)	1 (1.8)	3 (17.6)	1 (1.4)*
18877C>T	20 (22.0)	37 (17.5)	8 (22.9)	12 (21.4)	3 (17.6)	17 (23.0)
Spike, 23403A>G	74 (81.3)	181 (85.8)	28 (80.0)	48 (85.7)	10 (58.8)	64 (86.5)*
ORF3a						
25563G>T	56 (61.5)	145 (68.7)	22 (62.9)	36 (64.3)	9 (52.9)	47 (63.5)
26144G>T	4 (4.4)	10 (4.7)	3 (8.6)	1 (1.8)	3 (17.6)	1 (1.4)*
Matrix, 26625C>T	3 (3.3)	22 (10.4)*	2 (5.7)	2 (3.6)	1 (5.9)	2 (2.7)
ORF8, 27964C>T	0 (0.0)	13 (6.2)*	0 (0.0)	0 (0.0)	0 (0.0)*	0 (0.0)

© 2021 Esper FP et al. *JAMA Network Open.*

eFigure 3. SARS-CoV-2 Viral Load by GSAID Clade and Clade Group.

Viral load from initial SARS-COV-2 sample was derived from the Ct value using a standard curve and compared. Ordinary One way ANOVA was performed for GISAID clades while Student T-test was utilized for comparison between clade groups; both at a significance level of 0.05.

eFigure 3. SARS-CoV-2 Viral load by GSAID Clade and Clade Group

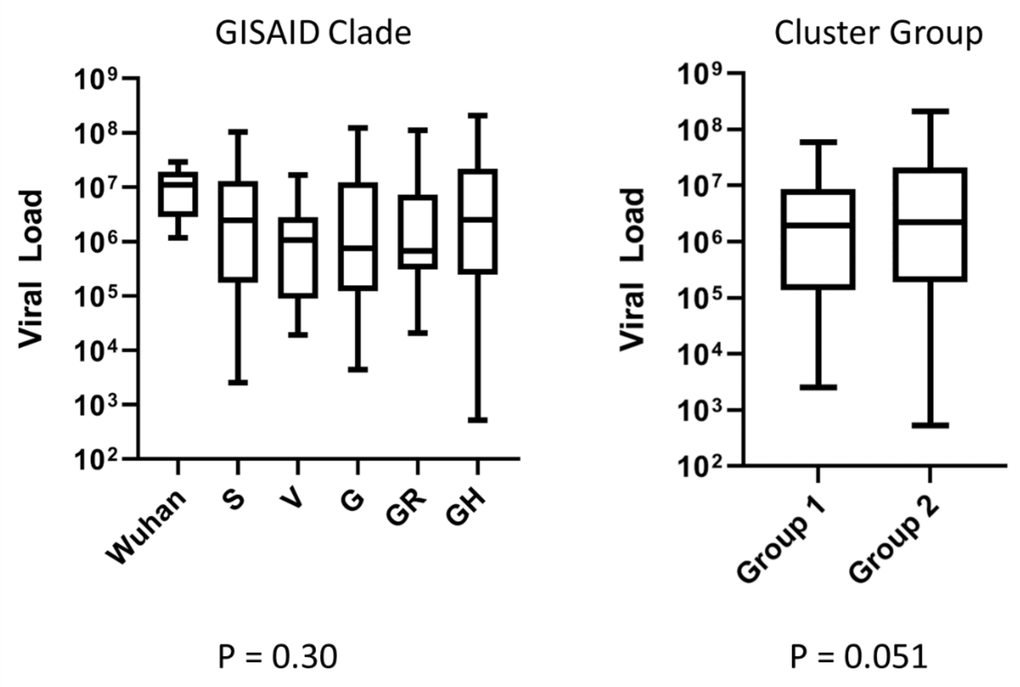

© 2021 Esper FP et al. *JAMA Network Open.*

eFigure 4. Specific Laboratory Abnormalities Among Selected SARS-CoV-2 Variants. Box and Whiskers plot displaying 1st through 99th percentile laboratory results among patients infected with specific SARS-COV-2 clades. P-values via Ordinary One way ANOVA at a significance level of 0.05 are displayed.

eFigure 4. Specific Laboratory Abnormalities Among Selected SARS-CoV-2 Variants

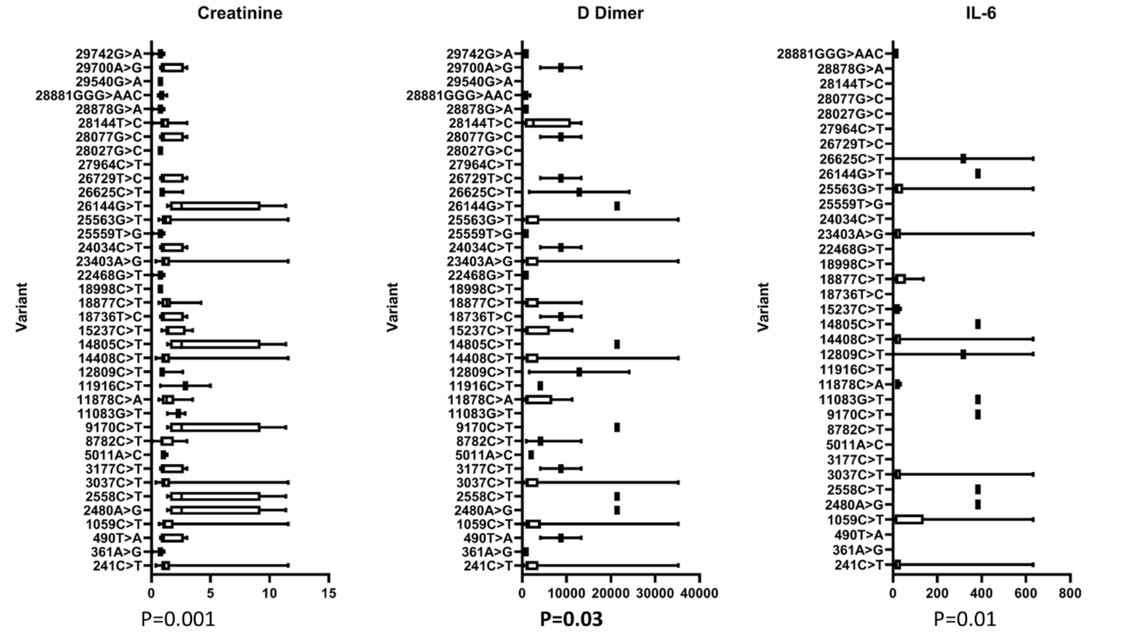

© 2021 Esper FP et al. *JAMA Network Open.*

eFigure 5. Comparison of Laboratory Abnormalities Among Different SARS-CoV-2 Clade Groups. Box and Whiskers plot displaying 1st through 99th percentile laboratory results among patients infected with specific SARS-COV-2 clades. P-values were obtained from Student T test.

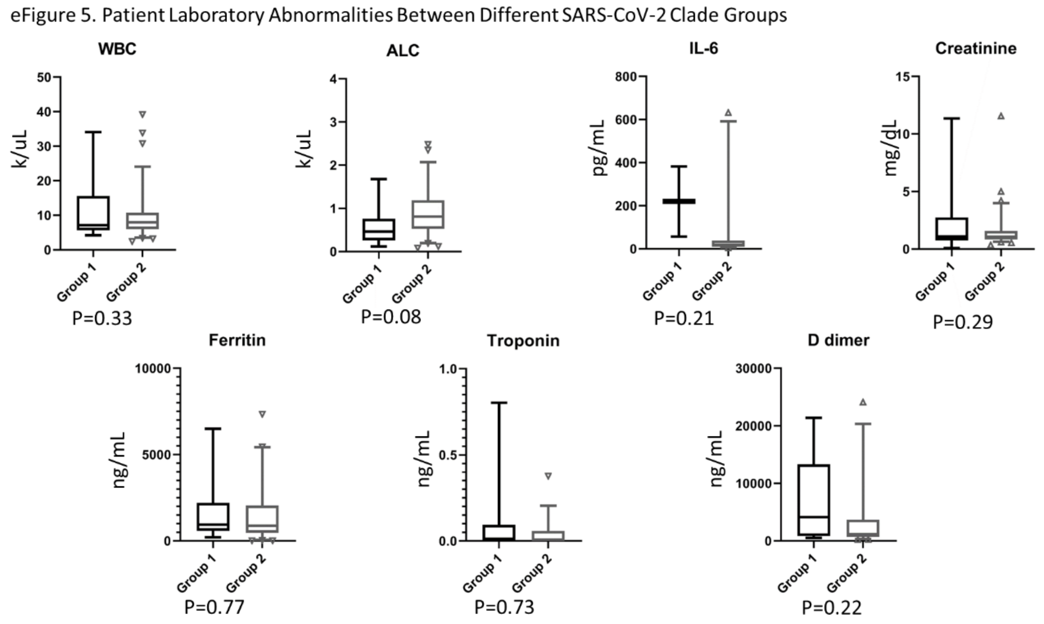

© 2021 Esper FP et al. *JAMA Network Open.*
